# Sickle Cell Anaemia among Tharu Population Visiting the Outpatient Department of General Medicine of a Secondary Care Centre: A Descriptive Cross-sectional Study

**DOI:** 10.31729/jnma.7651

**Published:** 2022-09-30

**Authors:** Subhash Pandey, Nitesh Shrestha

**Affiliations:** 1 Department of General Medicine, Bardiya Hospital, Gulariya, Bardiya, Nepal; 2 Department of General Medicine, Kaushalya Memorial Hospital, Kohalpur, Banke, Nepal

**Keywords:** *epidemiology*, *sickle cell disease*, *sickle cell trait*

## Abstract

**Introduction:**

Sickle cell anaemia is a global health issue in which a mutation of the 3-globin gene changes normal haemoglobin in sickle-shaped red blood cells. The objective of this study was to find out the prevalence of sickle cell anaemia among the Tharu population visiting the outpatient Department of General Medicine in a secondary care centre.

**Methods:**

A descriptive cross-sectional study was conducted among the Tharu population in the Department of General Medicine of a secondary care centre from 10 January 2020 to 10 June 2022. Ethical approval was taken from the Institutional Review Committee of the same institute (Reference number: 590/2076-077). A convenience sampling technique was used. Point estimate and 95% Confidence Interval were calculated.

**Results:**

Out of 409 patients, 60 (14.67%) (11.24-18.10, 95% Confidence Interval) had sickle cell anaemia. Among them, 45 (75%) patients had sickle cell trait and 15 (25%) patients had sickle cell disease.

**Conclusions:**

The prevalence of sickle cell anaemia was higher when compared to other studies conducted in similar settings. The government needs to emphasise more effort in diagnosing cases as well as increasing the testing centre.

## INTRODUCTION

Sickle cell anaemia is a common disease condition. It is a non-communicable disease which is characterised by episodic acute illness, debilitating pain crises, chronic anaemia and progressive multi-organ damage.^1,2^ Sickle cell trait carries only one copy of the altered haemoglobin gene and rarely has any clinical symptoms related to the disease while sickle cell disease carries two copies of the altered haemoglobin gene which destroyed red blood cells (RBC) rapidly and patients have chronic, severe anaemia, or low haemoglobin levels and have clinical symptoms.^3^

The first case of sickle cell anaemia was reported in 2003 in Nepal.^4^ There are little nationwide epidemiological data in Nepal regarding how many people are suffering from this disease and how many of them are carriers.

This study aimed to find out the prevalence of sickle cell anaemia among Tharu population visiting the outpatient Department of General Medicine in a secondary care centre.

## METHODS

A descriptive cross-sectional study was conducted in the Department of General Medicine of Bardiya Hospital, a secondary care centre from 10 January 2020 to 10 June 2022. Ethical approval was taken from the Institutional Review Committee of the same hospital (Reference number: 590/2076-077). Patients from the Tharu community to the outpatient department were included in our study. Informed consent was taken from the included participants. A convenience sampling was done. The sample size was calculated using the following formula:


$$\begin{array}{ll} \text{n} & ={\text{Z}}^{2}\times \frac{\text{p}\times \text{q}}{{\text{e}}^{\text{2}}}\\ & =1.96^{2}\times \frac{0.5\times 0.5}{0.05^{2}}\\ & =385 \end{array}$$

Where,

n= minimum required sample size
Z= 1.96 at 95% Confidence Interval (CI)
p= prevalence taken as 50% for maximum sample size calculation
q= 1-p
e= margin of error, 5%

The minimum required sample size was 384. However, a total of 409 patients were taken for the study.

Sickle cell anaemia was diagnosed by the presence of the haemoglobin 'S' band and by the presence of haemoglobin 'A' on Hb electrophoresis. Sickle cell traits were those patients whose electrophoresis showed the presence of both haemoglobin 'A' band and haemoglobin 'S' band-HbAS genotype and homozygous sickle cell disease were patients whose electrophoresis showed the presence of haemoglobin 'S' band with or without haemoglobin 'F' band-HbSS genotype. In our hospital, we diagnose sickle cell by performing High-performance liquid chromatography (HPLC) on haemoglobin (Hb) combined with clinical symptoms.^5^ It was performed by the BIO-RAD D-10 haemoglobin Testing System, BioRad Laboratories, Inc. (Serial Number: DJ9L454478) instrument in all patients.^4,5^ The results were recorded in the proforma.

Data were collected and entered in Microsoft Excel 2007 and analysed in the IBM SPSS Statistics 25.0. Point estimate and 95% CI were calculated.

## RESULTS

Out of 409 outpatients, 60 (14.67%) (11.24-18.10, 95% CI) had sickle cell anaemia. Among them, 45 (75%) patients had sickle cell trait and 15 (25%) patients had sickle cell disease.

A total of 30 (66.67%) were females and 15 (33.33%) were males among patients with sickle cell trait and the ratio of females to males was 2:1. Patients in the age group 21-40 years were 21 (46.67%) and >71 years were two (4.44%) (Table 1).

**Table 1:** Age and gender-wise distribution of patients with sickle cell trait among the Tharu population (n= 45).

Age group (years)	Females n (%)	Males n (%)
<20	8 (17.78)	5 (11.11)
21-40	13 (28.89)	8 (17.78)
41-60	5 (11.11)	1 (2.22)
61-70	2 (4.44)	1 (2.22)
>71	2 (4.44)	-
Total	30 (66.67)	15 (33.33)

Similarly, among patients with sickle cell disease, five(33.33%) were females and 10 (66.67%) were malesand the ratio of female to male was 1:2. Patients in theage group <20 and 21-40 years were eight (53.33%)and six (40%) respectively (Table 2).

**Table 2:** Age and gender-wise distribution of patients with sickle cell disease among the Tharu population (n= 15).

Age group (years)	Females n (%)	Males n (%)
<20	2 (13.33)	6 (40.00)
21-40	2 (13.33)	4 (26.67)
41-60	1 (6.67)	-
61-70	-	-
>71	-	-
Total	5 (33.33)	10 (66.67)

## DISCUSSION

In our study, 14.67% of the patients had sickle cell anaemia. Sickle cell anaemia is a common problem that leads patients to visit OPD. The greatest burden of this disease is seen in Asia, Africa and the Middle East.^6,7^ In a study performed on the Tharu people of Dang (Nepal) 4.5% were confirmed sickle cell positive.^2^ Bardiya lies in Lumbini province in midwestern Nepal where the majority of the population belongs to the Tharu community. In a study from India, sickle cell trait occurred in 9.30-10.6% and the disease in 0.21-0.6%^8,9^ While study from Saudi Arabia founds that 0.32% had sickle cell trait.^7^ In one study in India the ratio of male to female in sickle cell trait and sickle cell disease was 1:1,^8^ however in our study, sickle cell trait female to male ratio was 2:1 and in sickle cell disease female to male ratio was 1:2. Our study showed that the prevalence of sickle cell anaemia in women was slightly higher than in men, with the female to male ratio of 1.2:1, which is higher.

The most common age group of sickle cell trait in our study was 21-40 years at 46.67%. In our study, the lowest age group of sickle cell trait was in >71 years which is 4.44% and only found in females. In one study from India, it was found that a maximum number of sickle cell traits were found in 21-25 years of age,^9^ which is similar to that study. On the other hand, the most common age group of sickle cell disease in our study was <20 and 21-40 years seen in 53.33% and 40% respectively, and in the age group above 60 years, we did not find sickle cell disease. In one study from India,^9^ and Uganda it was found that a maximum number of cases were found in ages 0-5 years.^10^ In our study most number of sickle cell diseases were below 20 years.

The Nepalese government recently announced free treatment for individuals with an established diagnosis of sickle cell.^2,5^ That means before diagnosing cases patients need to pay from their pocket which is expensive and not everyone can afford it. In Bardiya, only our institution had a testing centre where Hb electrophoresis for the sickle cell can be performed throughout the year which is funded by the Province Government, Lumbini Province, Ministry of Health and Population Family Welfare (MoHPFW) and it is free of cost. Although there are few private laboratories which also perform the test they charge high amounts so locals cannot afford that is why in most cases patients remain undiagnosed or unrecognised.

The sample is specifically limited to the Tharu community patients attending OPD of Bardiya Hospital located in mid-western Nepal. Findings are limited because they are based on a limited population at one site due to fewer testing centres for sickle cell anaemia. Future studies in a community setting with a higher sample size are recommended.

## CONCLUSIONS

The prevalence of sickle cell anaemia among the Tharu population was higher when compared to other studies done in similar settings. The government of Nepal needs to emphasise more effort in diagnosing cases as well as increase testing centres and increase disease awareness regarding marriage, family genetic counselling and treatment among Tharu people and also needs to improve access to health resources sustainably.
